# Reinforcement Learning-Enabled Control and Design of Rigid-Link Robotic Fish: A Comprehensive Review

**DOI:** 10.3390/s26030996

**Published:** 2026-02-03

**Authors:** Nhat Dinh, Darion Vosbein, Yuehua Wang, Qingsong Cui

**Affiliations:** 1 Smart Devices and Intelligent Systems Laboratory, New Mexico Institute of Mining and Technology, Socorro, NM 87801, USA; 2 Department of Computer Science, East Texas A&M University, Commerce, TX 75428, USA

**Keywords:** rigid link fish robots, reinforcement learning, Q-learning, deep Q-network, deep deterministic policy gradient

## Abstract

With the rising demand for maritime surveys of infrastructure, energy resources, and environmental conditions, autonomous robotic fish have emerged as a promising solution with their biomimetic propulsion, agile motion, efficiency, and capacity for underwater inspection, monitoring, data collection, and exploration tasks in complex aquatic environments. Inspired by fish spines, rigid-link fish robots (RLFRs), a category of robotic fish, are widely utilized in robotics research and applications. Their rigid, actuated joints enable them to reproduce the undulatory locomotion and high maneuverability of biological fishes, while the modular nature of rigid links between joints makes them cost-effective and easy to assemble. This review examines and presents recent approaches and advancements in the field of structural design, as well as Reinforcement learning (RL)-enabled controls with sensors and actuators. Existing designs are classified by joint configuration, with key structural, material, fabrication, and propulsion considerations summarized. The review highlights the use of Q-learning, Deep Q-Network (DQN), and Deep Deterministic Policy Gradient (DDPG) algorithms for RLFR controllers, showing their impact on adaptability, motion control, and learning in dynamic hydrodynamic conditions. Technical challenges—including unstructured environments and complex fluid–body interactions—are discussed, along with future directions. This review aims to clarify current progress and identify technological gaps for advancing rigid-link robotic fish.

## 1. Introduction

Over the past two decades, biomimetic underwater vehicles (BUVs)/autonomous robotic fishes have garnered significant attention for their ability to mimic the swimming mechanisms of marine animals, enabling environmental monitoring, marine exploration, surveillance, and research on aquatic ecosystems. Biological fish have developed fast, quiet, and efficient means of underwater locomotion. To ensure biomimetic robots work reliably in marine environments, their design, structure, and control strategies need to incorporate these abilities. There are four major categories of biomimetic robots: Rigid-Link Fish Robots (RLFRs) [1,2,3,4], soft or continuum robotic fish [5,6], tail-actuated robotic fish [7,8], and multi-joint robotic [9,10].

RLFRs and soft robotic fish have gained recognition as a promising class of biomimetic systems that enable the replication of real fish’s swimming mechanics through rigid links on their bodies. These robots achieve forward swimming and turning by mimicking the caudal fin (BCF) propulsion of natural swimmers, which involves sequential oscillations along the body [11,12]. Soft robotic fishes are naturally flexible, can change shape easily, and could store and recover energy passively while swimming. Their continuum bodies allow them to naturally adapt to complex flow conditions, potentially making them more durable and energy-efficient than rigid-link designs [5,13,14]. However, these advantages come at the expense of increased modeling and control complexity. Soft robots often exhibit high-dimensional, continuous deformation states, strong material nonlinearities, and limited direct sensing of their internal shape. These factors make it harder to estimate their state and to achieve stable reinforcement learning training. On the other hand, rigid-link fish robots have clear, well-defined joint states, accurate actuation, and repeated kinematics. This makes it easier to show the relationship between states and actions and create more reliable reinforcement learning rewards. Compared with soft or other flexible fish robots, RLFRs can also accommodate diverse actuation mechanisms (servo, DC motor, cable driven, or hybrid) and be a better platform for applying Reinforcement Learning (RL) due to their modular architecture, allowing precisely controlled torque and angle of each joint, and mechanical repeatability minimizes uncertainties caused by material deformation.

From the early single-joint fish robot to later multiple-joint robots sharing a rigid head that contains electric parts connected to a flexible body or caudal fin [15,16,17], the development of RLFRs is understood as an increase in the number of active joints in the body and the development of control strategies for RLFRs reflects an increasing level of biological fidelity and computational sophistication. Early single-joint prototypes typically relied on open-loop control, where preprogrammed sinusoidal signals drove the caudal fin without feedback. Although this approach enabled stable oscillatory motion, it lacked adaptability to environmental disturbances. Consequently, closed-loop control strategies incorporating sensor feedback, such as inertial measurement units (IMUs), encoders, or flow sensors, were introduced to improve trajectory tracking and maneuverability. Despite these improvements, closed-loop control remains challenging due to the nonlinear hydrodynamic coupling between joints, phase lag effects, and sensitivity to parameter tuning—which can interrupt stable swimming behavior [18].

To generate more natural rhythmic locomotion, central pattern generator (CPG) models have been widely adopted, using coupled nonlinear oscillators to produce coordinated traveling waves along the robotic body. CPG-based controllers enable smooth multi-joint motion with relatively low computational cost [17,19]. However, both CPG-based and traditional control methods depend heavily on predefined parameters and simplified dynamic models, limiting their adaptability under unsteady hydrodynamic conditions, often necessitating manual tuning of oscillator gains, phase couplings, and feedback coefficients.

In recent years, artificial intelligence (AI), Reinforcement Learning (RL), and data-driven intelligent control have experienced significant growth and are being applied in robotic systems. Theoretically, RL is a branch of machine learning (ML) in which agents learn optimal policies through trial-and-error interactions with the environment. Common RL algorithms applied to RLFRs include Q-learning, Deep Q-Networks (DQN), and Deep Deterministic Policy Gradient (DDPG) methods [20,21]. Using sensor-derived states and carefully designed reward functions, RL-based controllers enable robotic fish to autonomously learn swimming, turning, depth control, and obstacle avoidance behaviors [22,23]. Despite these advances, several challenges remain. Sim-to-real transfer remains challenging due to discrepancies between simplified hydrodynamic models and the complex dynamics of the real world. RL training often suffers from low sample efficiency, as underwater experimentation is costly and time-consuming. Real-time deployment is constrained by onboard computational limitations, energy capacity, and control latency in wet environments. Moreover, RLFRs must operate robustly under continuous disturbances such as turbulence, sensor noise, and varying water pressure, while hardware constraints restrict continuous online learning and adaptation.

Although research on RL-enabled robotic fish has increased rapidly, most existing studies focus on improving performance for specific tasks under fixed mechanical designs. As a result, reinforcement learning is often used as a tuning tool rather than as a driver of fundamental change. A key open question remains: how can reinforcement learning be used to achieve qualitatively new capabilities in robotic fish, such as higher adaptability, robustness to uncertainty, and scalability across different designs and environments? Addressing this question requires looking beyond individual algorithms and examining how mechanical design, sensing, and learning interact at the system level.

In this study, we follow the Preferred Reporting Items for Systematic Reviews and Meta-Analyses (PRISMA) guidelines to conduct a systematic literature review examining the combined development of mechanical design and learning-based control strategies in rigid-link fish robots (RLFRs) over the past 20 years. By studying existing research based on joint architecture, from single-joint to multi-joint designs and control strategies, the primary contribution of this review lies in its system-level synthesis that explores how increasing mechanical articulation and reinforcement learning-based control fundamentally reshape controllability, sensing requirements, and achievable control objectives. Rather than serving only as a descriptive survey, this review aims to guide future progress by identifying where current approaches fall short and how these limitations can be overcome. By comparing robotic fish across different joint architectures and learning strategies, this review highlights several promising directions, including learning methods that adapt to robot morphology, reusable swimming behaviors that can be transferred across tasks, learning frameworks that better account for real fluid dynamics, and reinforcement learning approaches that scale to multiple cooperating robots. Together, these directions illustrate how reinforcement learning can move beyond incremental control improvements and enable meaningful breakthroughs in rigid-link robotic fish. As articulation complexity and fluid–structure interactions increase, many traditional model-based controllers and manually designed learning methods become unsuitable or increasingly limited, particularly in unstructured underwater environments. Within this context, reinforcement learning is considered not merely an isolated algorithmic improvement, but a data-centric control methodology that arises from increasing system complexity and unpredictability. Through a cross-cutting synthesis of representative RL-enabled RLFR studies, this review clarifies common design patterns and algorithmic trade-offs and identifies open challenges and research gaps that will guide future studies, rather than focusing on any existing or new algorithms, hardware designs, or implementations.

**Figure 1:**
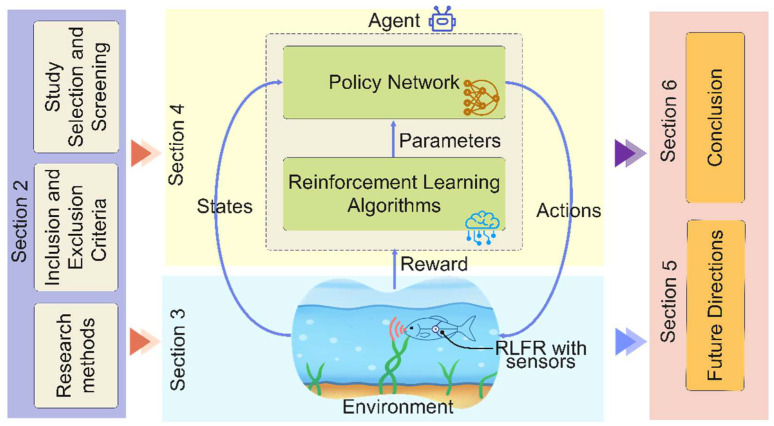
Logical framework of our review with PRISMA principles (in Section 2), RLFRs (in Section 3), and reinforcement learning algorithms (in Section 4) with future research directions discussed in Section 5 and conclusion in Section 6.

The remainder of this review focuses on the development of prototypes for RLFRs, and the design of RL algorithms for the controller is organized as shown in Figure 1. Section 2 describes the methodology used in this review. Section 3 examines RLFR designs, including body architecture, materials, and propulsion strategies. Section 4 focuses on RL algorithms, including Q-learning, DQN, and DDPG, applied to RLFRs. Section 5 discusses the challenges and future directions of RL in the field of RLFRs. Finally, Section 6 concludes this review.

## 2. Review Methodology

### 2.1. Research Methods

PRISMA guidelines were followed to systematically locate and examine previous studies, ensuring the literature survey is both organized and repeatable. Six major scientific databases were selected: ProQuest, Scopus, Web of Science (WoS), ScienceDirect, IEEE Xplore, and Google Scholar. These databases include journals and conference proceedings pertinent to robotics, control systems, mechatronics, bioinspired engineering, and underwater robotics. They have been widely utilized in previous systematic reviews [11,15,16,17,18,24] within the engineering and biomedical fields. This enables a comprehensive examination of the state of the art in RLFRs, with a particular emphasis on mechanical design, sensor integration, and reinforcement learning-based control strategies.

The search was performed based on combinations of terms related to robotic fish morphology, actuation, and learning-based control, including “biomimetic robotic fish”, “rigid-link”, “multi-joint”, “reinforcement learning”, “DDPG”, etc. Representative Boolean search strings were then formulated, including (“robotic fish” OR “biomimetic fish robot”) AND (“reinforcement learning” OR “deep reinforcement learning”) and (“rigid-link” OR “multi-joint”) AND (“fish robot” AND “control”). Additionally, to reduce omission bias, backward and forward citation tracking was performed on highly cited articles and recent review papers.

### 2.2. Inclusion and Exclusion Criteria

The reviewed literature was limited to scientific articles, peer-reviewed journal articles, and refereed conference papers written in English and published between 2004 and 2025. Earlier works were included as necessary to establish foundational concepts in robotic fish design and classical control theory. Case studies and experimental validation papers were included when they provided substantial technical insight into design or control performance on robotic fish or fish-inspired underwater robots. It was also necessary to address mechanical designs involving rigid linkages or multiple joints, as well as to examine control strategies that incorporate reinforcement learning or other advanced learning-based approaches. Both studies that used simulations and those that reported experimental results, as well as those that combined simulations and experiments, were considered for inclusion.

Studies were excluded if they focused only on biological aspects without any robotic design or application, or if they investigated general autonomous underwater vehicles (AUVs) without fish-like movement. Additionally, works that focused solely on soft robotic systems, without articulated or rigid-link structures, were excluded. Publications that weren’t peer-reviewed, such as abstracts, technical reports, and studies that lacked sufficient methodological or technical detail, were also excluded from the review.

### 2.3. Study Selection and Screening

**Figure 2:**
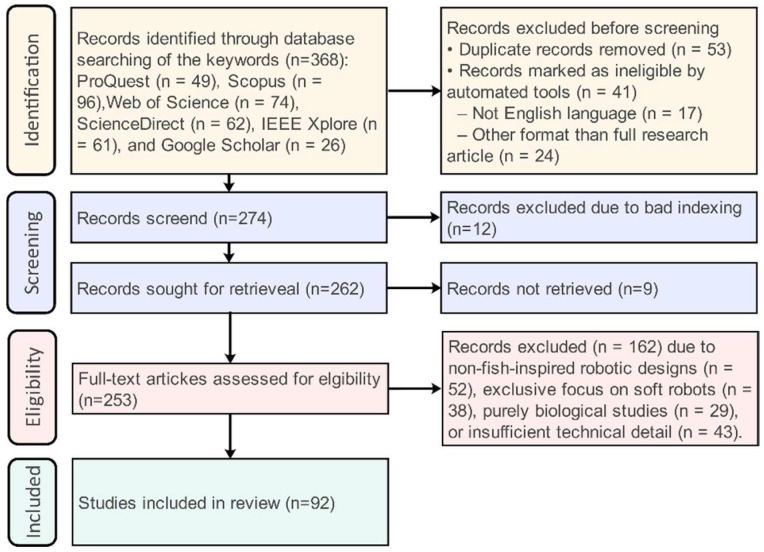
PRISMA flow diagram of the selected records.

**Figure 3:**
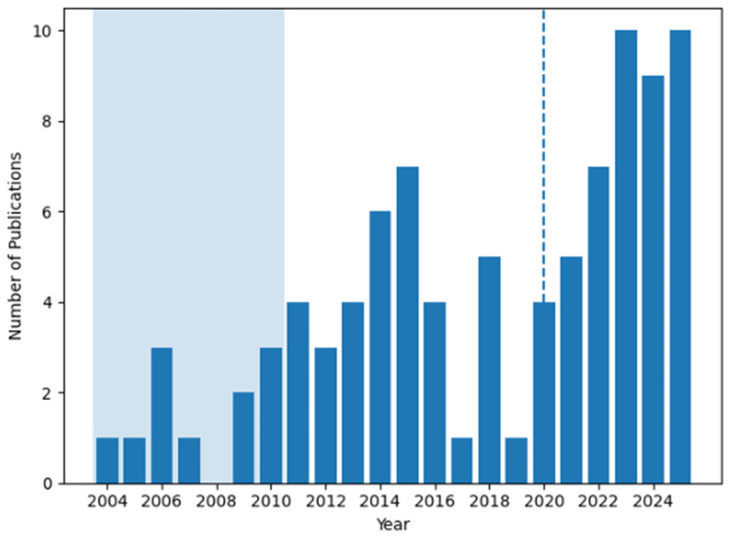
Distribution of publications on rigid-link robotic fish by year (2004–2025).

A total of approximately 368 records were initially identified through database searches, including about 96 records from Scopus, 74 from Web of Science, 49 from ProQuest, 61 from IEEE Xplore, 62 from ScienceDirect, and 26 from Google Scholar. After duplicate removal and relevance screening, 92 publications were included in this review (see Figure 2). Figure 3 shows the distribution of publications on rigid-link robotic fish by year (2004–2025). The shaded area represents the early research period, from 2004 to 2010. The dashed vertical line in 2020 marks a shift toward rapid growth, driven by advances in learning-based control methods. For each selected study, relevant technical and methodological information was extracted and organized, covering mechanical architecture, actuation and sensing strategies, control frameworks and learning algorithms, training environments, target tasks, and validation methods. The collected data were summarized and categorized according to the literature, based on the design evolution from single joints to multiple joints and RL-based control strategies, facilitating a structured comparison across the reviewed studies.

## 3. Design of Rigid Links Fish Robots

**Figure 4:**
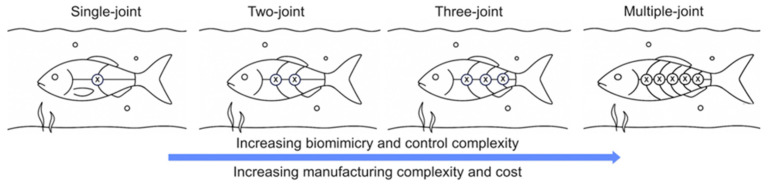
RLFR classification by articulation from single-joint (**left**) to multiple-joint (**right**).

The robotic fish field encompasses a variety of designs, but rigid-link fish robots (RLFRs) offer structural simplicity and controllable motion while maintaining high maneuverability, flexibility, and excellent swimming performance. Inspired by nature, where real fish propel themselves by undulating their bodies or tails to accelerate the surrounding fluid, RLFRs mimic this morphology through rigid linkage mechanisms. To better understand their structural and functional diversity, RLFRs can be classified by the number of joints, as shown in Figure 4. This classification shows how structural components, materials, joint types, and motion strategies contribute to RLFR performance in underwater environments. A comparative comparison is summarized in Table 1.

### 3.1. Single-Joint Structure

**Figure 5:**
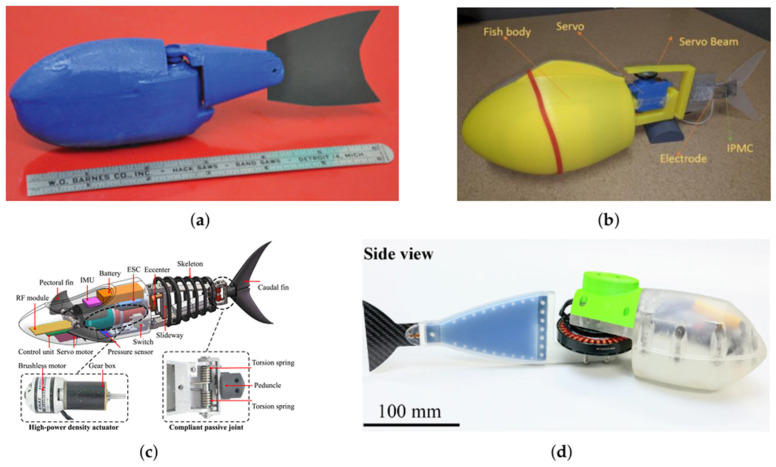
Typical designs of RLFRs with a single joint: (**a**) Biomimetic robotic fish designed by KopmanandPorfiri [1]; (**b**) Robotic fish proposed by Chen et al. [31]; and (**c**) Mechanical structure created by Chen et al. [33]; (**d**) the side view of robotic fish developed by Iguchi et al. [13].

Bio-inspired rigid designs with a single joint on the fish body represent the simplest RLFRs configuration, offering low cost, structural simplicity, and ease of control. Most single-joint fish robots use an active joint to connect the rigid head housing electronics (e.g., actuators, power sources, sensors), and a tail fin generates periodic oscillations that produce forward propulsion. For instance, Kopman and Porfiri [1] developed a single rigid-link robotic fish actuated by a servo motor, as shown in Figure 5a. The robot is composed of an acrylonitrile butadiene styrene (ABS) plastic body and a compliant Mylar tail, which connects through a Traxxas 2065 servomotor (Traxxas, McKinney, TX, USA) to achieve a sinusoidal angular displacement between the body and the tail. These oscillations cause the compliant caudal fin to bend, generating forward thrust and a backward water flow. The shape of the caudal fin can be altered to optimize thrust production, and the oscillation frequency and amplitude were wirelessly controlled via an Arduino Pro Mini microcontroller (Arduino SA, Ivrea, Italy).

RLFRs primarily replicate carangiform or subcarangiform swimming patterns, which are distinguished by movement concentrated in the posterior portion of the body while the anterior segment remains relatively rigid. This approach minimizes unnecessary head oscillation, thereby decreasing hydrodynamic drag and enhancing energy efficiency.

The actuation of these robotic fish is typically achieved using regular servo motors or compact DC motors. This setup allows the robots to generate robust forward thrust while maintaining a simple mechanical structure. For instance, the iSplash-MICRO robotic fish, designed by Clapham and Hu [34], employs a carangiform swimming mode and features pronounced dorsal and pelvic fins to enhance lateral stability. Its propulsion system utilizes a crankshaft-linkage mechanism to convert the rotary motion of a miniature electric motor into oscillations of the tail. As a result, the robot can reach a maximum swimming speed of 10.4 body lengths per second (0.52 m/s) while consuming only 0.8 W of power.

Although these single-joint designs may appear mechanically straightforward, their scientific significance lies in the transparent relationship between control inputs and hydrodynamic responses. Unlike multi-joint or soft-bodied fish robots, which rely on complex, distributed body deformations for propulsion, single-joint RLFRs offer a clear platform for experimentally validating theoretical models of swimming and for testing new learning algorithms. This direct correspondence between input and output makes single-joint RLFRs particularly valuable for research focused on underwater locomotion and control.

Body Caudal Fin (BCF) propulsion is integrated into single-joint RLFRs to achieve higher swimming efficiency and maneuverability. In BCF propulsion, the rigid anterior body remains nearly stationary, while the posterior tail and caudal fin perform oscillatory movements that generate thrust. Servo and DC motors are used to generate high torque flapping motion. Some models, however, have developed Ionic Polymer–Metal Composite (IPMC) actuators to provide flexibility and two-dimensional maneuverability, as illustrated in Figure 5b. IPMCs are soft electroactive materials composed of ion-exchange membranes coated with metal electrodes, enabling low-voltage actuation through ion migration-induced bending deformation [28,29,30,31,32]. However, compared to rigid-link actuation, IPMC-based systems often exhibit lower thrust density, limited operational bandwidth, and reduced control authority at high speeds or high loads. These limitations highlight an important trade-off between adaptability and propulsion performance when comparing soft actuators with rigid-link mechanisms.

Chen et al. [33] developed a high-performance single-joint RLFR using a high-power-density brushless DC motor (EMAX XA2212, 980 kV, Guangdong, China) combined with a GP22C planetary gearbox (maxon motor ag, Sachseln, Switzerland), as shown in Figure 5c. An eccentric crank–slider mechanism converts continuous motor rotation into lateral oscillations of the caudal peduncle. A compliant passive joint using symmetrical torsion springs was introduced between the posterior body and caudal fin to store and release elastic energy during the tail-beat cycle. This compliant configuration increased steady swimming speed from 2.6 to 3.8 body lengths per second (BL/s) and achieved a maximum instantaneous speed of 4.3 BL/s. In addition, Iguchi et al. [13] proposed a direct-drive (DD) single-joint robotic fish, illustrated in Figure 5d, in which a brushless DC motor is directly coupled to a flexible silicone–carbon fiber body. The DD mechanism reduces mechanical losses and enables high-bandwidth actuation, achieving a peak swimming speed of 6.3 body lengths per second and a maximum thrust of 63.2 N at resonance.

Given their mechanical simplicity, single-joint RLFRs serve as practical and strategically important platforms for applying reinforcement learning-based control. In single-joint designs, the robot’s swimming behavior is governed by a small and physically intuitive set of control variables, such as tail-beat frequency, oscillation amplitude, or motor current. Each of these parameters has a direct and observable influence on thrust generation and swimming stability. As a result, the learning agent can more easily associate control actions with hydrodynamic outcomes, reducing trial-and-error complexity during training and enabling faster convergence. Moreover, the strong, predictable coupling between actuation and propulsion in single-joint RLFRs enables the definition of reward functions in a physically meaningful manner. Performance objectives such as forward velocity, energy efficiency, or heading stability can be measured directly and reliably, enabling the experimental validation of learned policies. In contrast, multi-joint or soft-bodied robotic fish exhibit highly nonlinear body deformations and complex fluid–structure interactions, in which identical control inputs may yield inconsistent swimming responses, complicating both policy learning and interpretation.

Although soft robotic fish provide superior compliance and adaptability, their high-dimensional deformation states and material hysteresis introduce significant challenges for state estimation and policy generalization in RL. Single-joint rigid-link robots offer an effective compromise: they retain sufficient biological realism for meaningful swimming studies while maintaining a level of mechanical and dynamical simplicity that enables reinforcement learning to be controllable, interpretable, and experimentally repeatable. Overall, early single-joint RLFRs achieve strong swimming performance with minimal mechanical effort by concentrating actuation at a single posterior joint behind the rigid head. This configuration simplifies fabrication, wiring, sealing, and maintenance while still producing reliable, repeatable tail-beat dynamics. The widespread use of 3D-printed structures and commercial actuators allows consistent replication across studies, making these robots especially valuable as standardized experimental platforms for testing, comparing, and validating reinforcement learning-based control strategies in underwater environments.

### 3.2. Two-Joint Structure

**Figure 6:**
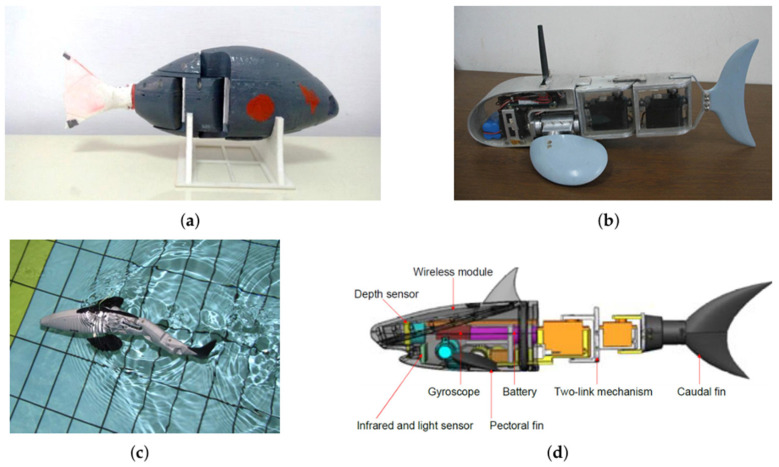
Typical designs of RLFRs with a two-joint: (**a**) robotic fish prototype created by Ay et al. [35]; (**b**) modular robotic fish designed by Hu et al. [36]; (**c**) fish-robot developed by Szymak et al. [37]; (**d**) mechanical configuration of the miniature robotic fish proposed by Yu et al. [3].

Due to limitations of maneuverability with single-joint structures, two-joint structure RLFRs are proposed to enhance agility, improve swimming efficiency, and facilitate more complex movement patterns. The structure of two-joint fish robots typically consists of three main rigid sections: a rigid head, a body segment, and a caudal fin, all of which are connected to each other by an active joint. For instance, Ay et al. [35] designed the i-RoF fish robot, as illustrated in Figure 6a, with an anterior rigid body, a two-link propulsive tail produced via 3D printing using PLA, and a flexible caudal fin made from silicone. Hu et al. [36] developed a module-based approach for the design of reconfigurable robotic fish, in which each module is waterproofed separately and can be added or removed as needed, as shown in Figure 6b. CyberFish, designed by Szymak et al. [37], can swim in the carangiform mode (see Figure 6c). In ref. [35], epoxy resin, O-rings, and grease seals were used at the active joints to enhance waterproofing. Additionally, a center-of-gravity (CoG) control mechanism uses a servo-driven sliding mass, enabling 3D maneuvering, including diving and surfacing. The propulsion system utilizes two servo motors to achieve a forward velocity of up to 0.85 body lengths per second (BL/s).

Yu et al. [3] developed a 35 cm miniature two-joint robotic fish, as shown in Figure 6d, comprising a lightweight composite structure that balances stiffness and flexibility for effective propulsion, and a shark-like body with a rigid head and a flexible tail. The design incorporates various sensors, including light, pressure, gyroscope, and infrared sensors, to enable environmental awareness and adaptive three-dimensional (3D) swimming control. The mechanical pectoral fins, driven by a gear-coupled servo system, provide stability, depth control, and turning. The propulsion system operates in a body–caudal fin (BCF) mode, enabling the robot to achieve swimming speeds of 0–0.5 m/s, powered by a 3400 mAh lithium battery. The robot maintains approximately one hour of continuous free swimming.

The propulsion mechanism structure of a two-joint fish robot is typically actuated by servo motors at active joints to generate swim motions of the caudal fin, which is similar to BCF locomotion. At the first joint (usually between the rigid head and the body), the servo motors create lateral oscillation, which serves as the primary source of propulsion initiation. The rotational motion from these motors is high-torque R/C or DC servomotors converted into a side-to-side angular displacement, which is the posterior segments and caudal fin to follow a traveling wave pattern that mimics real fish swimming. Using pulse-width modulation (PWM) signals from a microcontroller, the joint performs periodic sinusoidal motion within a ±20–30° range, establishing the body wave that propagates downstream.

Compared to single-joint models, two-joint RLFRs provide significantly improved agility and flexibility while maintaining manageable mechanical and control complexity. This first joint’s oscillation defines the overall tail-beat frequency, thrust direction, and swimming stability in two-joint rigid-link fish robots [34,36,38,39,40,41]. Meanwhile, the second active joint (located between the posterior body segment and the caudal fin or tail) serves as a wave amplifier, enhancing the body wave initiated by the first joint and then directly converting it into thrust. The servo motor drives this joint to create angular displacement synchronized with the first joint, but with a phase delay between −180° and 0°. The posterior servo often operates over a smaller angular range (±15–25°) to minimize drag while maintaining optimal thrust-to-power efficiency [37,42,43,44,45]. When combined with reinforcement learning, the second joint allows learning algorithms to shape body-wave propagation and thrust generation, rather than relying on fixed oscillation patterns as in a single joint. When transitioning from simulation to reality, two-joint designs with moderate mechanical complexity tend to yield lower modeling error than soft robots or those with many joints. Nevertheless, challenges related to waterproofing reliability and precise joint coordination remain open research problems. Overall, the two-joint architecture represents a balanced approach and offers a little advanced environment for applying RL algorithms.

### 3.3. Three-Joint Structure

Three-joint rigid-link fish robots (RLFRs) are designed to more accurately approximate the body-wave propagation observed in biological fish, bridging the gap between overly simplified single-joint systems and highly articulated or soft robotic designs. Unlike single-joint and two-joint structures, three-joint RLFRs enable spatial phase lag and amplitude modulation along the body, which are essential for generating carangiform and sub-carangiform swimming modes with improved thrust efficiency and maneuverability. From an RL perspective, a three-joint RLFR can provide a more stable learning environment than single- or two-joint designs, offering richer motion diversity without overwhelming control complexity. In comparison, single-joint robots, which limit RL to tuning parameters, a three-joint structure allows the agent to learn how body-wave shape, phase delay, and thrust generation interact along the body. The additional joint enables smoother wave propagation and finer gait modulation, while maintaining a compact action space. This balance allows RL policies to learn coordinated, biologically meaningful swimming strategies efficiently and robustly.

**Figure 7:**
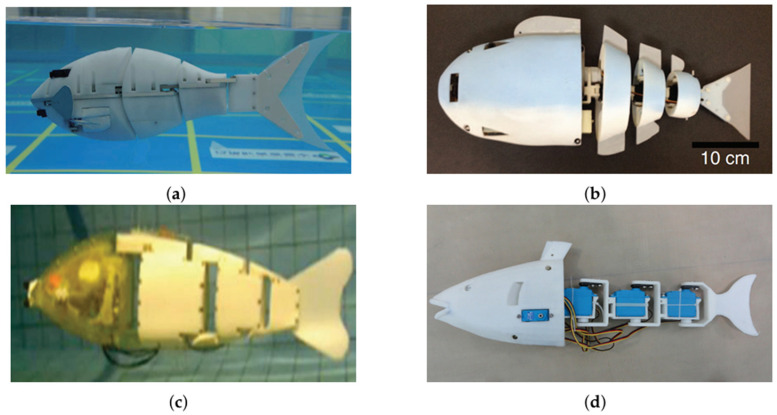
Typical designs of RLFRs with a three-joint structure: (**a**) robotic Fish ‘Ichthus V3.1’ developed by Yang et al. [4]; (**b**) side view of the robotic fish designed by PhamDuy et al. [46]; (**c**) Fibo II pro-posed by Na et al. [51]; (**d**) side view of the fish skeleton created by Solanki et al. [53].

Structurally, three-joint RLFRs are typically segmented into four main components: a rigid head, two intermediate body segments connected via active joints, and a posterior tail or caudal fin. Together, these components form a kinematic chain with three degrees of freedom, enabling coordinated undulatory motion along the body. Figure 7a illustrates the design of a three-joint RLFRs developed by Yang et al. [4] with segments such as head, bodies made from rigid polymer composites. These segments are connected to active joints, and each joint is actuated by a waterproof servo motor directly coupled to the link through mechanical couplers. Phamduy et al. [46], introduced a 46 cm-long fish-shaped robot featuring a rigid electronics-housing head and three Hitec HS-82MG servomotors (HiTec RCD, Poway, CA, USA) driving rigid body links connected to a flexible silicone caudal fin, as seen in Figure 7b. Lightweight polymers such as ABS, PLA, and Onyx composite are commonly used for the rigid segments and joints, primarily due to their compatibility with additive manufacturing. These materials allow rapid prototyping, high geometric accuracy, watertight integration, and buoyancy control through internal cavities. To emulate the compliant behavior of real fish, elastic materials such as silicone rubber and spring steel are incorporated into tail sections and fins, enabling passive bending and elastic energy storage during oscillation.

In terms of fabrication, three-joint RLFRs predominantly rely on 3D printing due to its low cost, ease of customization, and capacity to integrate internal wiring channels and buoyancy chambers. Precision components such as servo mounts, cranks, and slider mechanisms are often manufactured via laser cutting to ensure dimensional accuracy [47,48,49,50]. The primary propulsion mechanisms in three-joint RLFRs include servo motors, DC motor–gear couplings, and electromagnetic actuators. Servo motors remain the most widely adopted solution, with each servo functioning analogously to a vertebral joint. Coordinated actuation across joints generates a traveling body wave that closely resembles the locomotion of biological fish [51,52,53,54,55,56,57]. However, the relatively large size of servo motors limits miniaturization and increases inertial load. DC motor systems with gear transmission are commonly implemented when higher torque density is required. Jia et al. [58] employed compact, waterproof DC motors coupled with reduction gears to actuate the tail joints, enabling high-torque oscillatory motion and effective carangiform propulsion. Alternatively, Wang et al. [59] developed an electromagnetic propulsion system that generates torque through interactions between coil-induced magnetic fields and permanent magnets, enabling lightweight joints capable of high-frequency oscillation.

Overall, three-joint RLFRs achieve more agile maneuvering and swimming motions that more closely resemble those of real fish when compared with simpler single- or two-joint designs. Adding an extra joint also makes control more challenging: the joints must be precisely synchronized, small timing errors can degrade thrust, and uncoordinated motion can significantly increase energy consumption. Reinforcement learning is therefore well-suited to this setting, as it can learn coordinated joint behaviors through interaction, automatically adjusting timing, amplitude, and joint coupling to achieve efficient propulsion while maintaining stability and limiting unnecessary power use.

### 3.4. Multiple-Joint Structure

**Figure 8:**
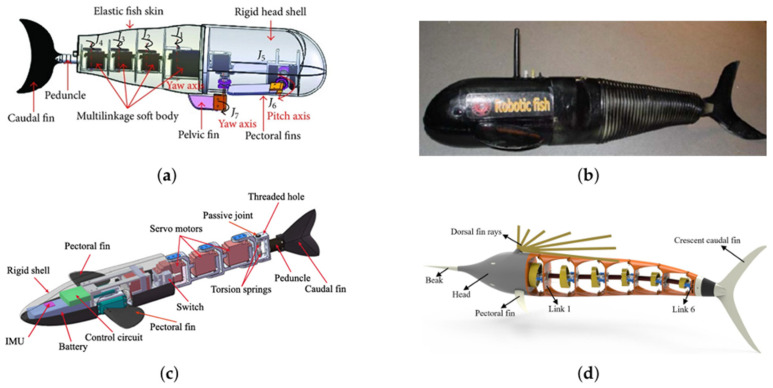
Typical designs of RLFRs with a multiple-joint structure: (**a**) prototype of multi-mode robotic fish developed by Yu et al. [62]; (**b**) 3D body structure of sailfish-like robot designed by Zhao et al. [63]; (**c**) 3D mechanical structure of the multi-joint robotic fish proposed by Chen et al. [10]; (**d**) mechanical drawing of a self-propelled robotic fish created by Han et al. [67].

The robotic fish with four or more joints, as shown in Figure 8, have emerged as a dominant trend in biomimetic underwater vehicle design. With only one-, two-, or three-joint structures, robotic fish can generate tail oscillation and basic turning maneuvers; however, body curvature is typically concentrated at a limited number of discrete joints. As a result, the swimming motion appears segmented, the body-wave envelope is poorly distributed, and the achievable turning radius remains relatively large. From a control perspective, these low-degree-of-freedom (DoF) designs limit the spatial resolution of body-wave modulation, thereby reducing the expressive control authority available to both classical controllers and RL agents. Instead, the multi-joint architectures can enable smoother curvature distribution, continuous traveling-wave propagation, and more faithful reproduction of carangiform and subcarangiform swimming modes [60,61]. The increased number of joints expands the action space in a structured, physically interpretable manner, allowing RL policies to coordinate amplitude gradients, inter-joint phase delays, and frequency coupling across the body rather than relying on a single global oscillation parameter.

A four-link modular body [62], shown in Figure 8a, consists of a rigid head connected to four servo-actuated joints, two pectoral fins, and one pelvic fin, each independently controlled by servo motors. The crescent-shaped caudal fin enhances thrust generation, while the pectoral fins (±180° rotation) enable forward, backward, and vertical motions. The pelvic fin, oscillating ±90°, functions as a rudder to improve lateral maneuverability and stability. This configuration illustrates how additional joints decouple propulsion, maneuvering, and stabilization functions, which is particularly advantageous for multi-objective RL formulations involving speed, heading control, and energy efficiency. Zhao et al. [63] proposed a four-link serial-chain robotic fish, as shown in Figure 8b, comprising a rigid fiberglass head, four servo-driven joints, and a flexible posterior body enclosed in a waterproof rubber sleeve. This backbone-like structure enables smooth body-wave propagation and natural carangiform locomotion. Concentrating the center of mass in the head section improves passive stability and reduces unwanted oscillations.

PLA and ABS polymers are commonly used for 3D-printed rigid head components, while aluminum or carbon-fiber composites are used for body links to ensure stiffness and precise torque transmission. These rigid modules are typically enclosed in a flexible waterproof skin to reduce drag and isolate servo actuators [64,65,66]. Compared to soft robots, which rely on distributed material compliance and complex fluid–structure interactions, rigid-link multi-joint designs offer more deterministic kinematics and more observable state variables; these are key advantages for data-efficient RL training and policy generalization. In contrast, Han et al. [67] introduced a sailfish-inspired robot (see Figure 8d) driven by a single motor, comprising six lightweight modular segments connected by cam–roller mechanisms and universal joints. This mechanical transmission enables smooth undulatory movement and eliminates distributed actuation, making waterproofing much simpler.

Most multi-joint rigid-link robotic fish rely on servo-based distributed actuation, in which each joint is independently driven and coordinated via phase-shifted sinusoidal inputs. This approach enables precise control of traveling waves and replicates biological carangiform swimming patterns [6,68,69,70]. However, tail-end servos must deliver higher torque to overcome hydrodynamic drag, often leading to amplitude attenuation at high frequencies. Chen et al. [10], as shown in Figure 8c, addressed this limitation by incorporating a passive compliant joint with torsion springs to mimic peduncle elasticity, achieving a swimming speed of 0.544 m/s (0.99 body lengths per second) at 2.4 Hz. Hybrid active–passive joint architectures such as this are particularly attractive for RL, as they reduce control effort while preserving sufficient action dimensionality for learning efficient propulsion strategies.

Overall, multi-joint rigid-link fish robots bridge the gap between monolithic rigid designs and intricate soft robotic systems. As discussed earlier, single- and two-joint robots are easy to control and useful for early RL testing, but their limited degrees of freedom prevent the learning of realistic, spatially distributed body-wave motion. Three-joint robots add flexibility, yet their discrete curvature still falls short of capturing the smooth undulation seen in real fish. Soft robots, on the other hand, offer excellent adaptability and energy efficiency, but their continuous deformation, material nonlinearities, and limited state observability make RL training difficult and computationally expensive. Robots equipped with four or more rigid joints achieve a practical equilibrium between complexity and control. This configuration provides sufficient degrees of freedom for RL algorithms to develop coordinated, biologically inspired swimming gaits, while maintaining rigid-body dynamics that facilitate modeling, sensing, and reliable transfer from simulation to experimental settings. Although challenges remain in waterproofing, torque transmission, and joint coordination, future designs that combine rigid links with variable-stiffness elements and sensory feedback could enable adaptive, energy-aware swimming without sacrificing RL tractability.

Several known limitations of classical RL methods are insufficiently discussed in prior RLFR studies, including Q-value overestimation in DQN, gradient instability and sensitivity to reward scaling in DDPG, and the challenges associated with sim-to-real transfer under complex fluid–structure interactions. These gaps necessitate a reassessment of existing approaches and the integration of modern RL algorithms and learning paradigms into future RLFR research. Accordingly, subsequent sections of this review not only analyze representative RL algorithms and their application to RLFRs but also critically examine their advantages, limitations, and transferability while identifying promising directions for next-generation learning-based control of robotic fish.

## 4. Reinforcement Learning-Based Control in RLFRs

The inherently high dimensionality of fluid–body interactions poses significant challenges for the formulation of accurate closed-form analytical models or solutions. In contrast to conventional analytical approaches, machine learning methods, particularly reinforcement learning, employ a trial-and-error framework in which an agent interacts with the environment and learns through a structured reward-based feedback system. Through this process, the robotic fish learns effective body movements that maximize propulsion efficiency, maneuverability, and stability, while also executing specific tasks [71,72].

**Figure 9:**
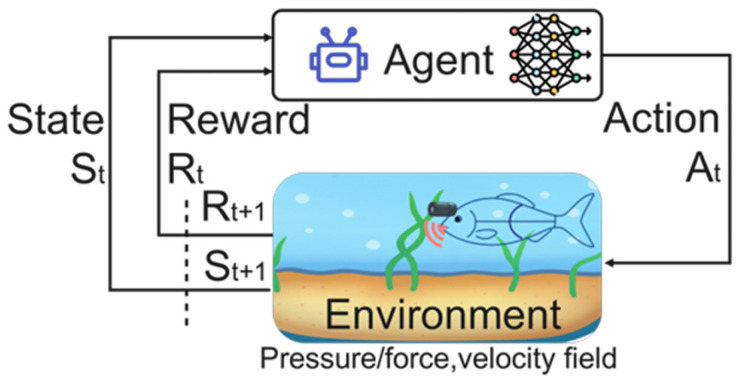
Markov Decision Process (MDP) of reinforcement learning.

A RL framework is normally formulated as a Markov Decision Process (MDP), defined as D ≡ (S, A, P, R), as illustrated in Figure 9. Here, s_t_ ∈ S denotes the state at time t, containing measurable sensory or simulated data (e.g., joint angles, pressure and force measurements, and the local flow velocity, and thrust). The action a_t_ ∈ A represents controllable variables such as joint torque, tail-beat frequency, or phase differences between body segments. The reward r_t_ ∈ R evaluates swimming performance, encouraging higher speed, improved energy efficiency, and accurate path tracking. The state transition probability P characterizes the dynamics of the environment, which may be unknown and highly nonlinear for fluid–body interaction systems. The agent serves as the fish controller, while the environment corresponds to the hydrodynamic conditions [14,24,71,72,73]. The RL process forms a continuous feedback loop between the agent and the environment. As shown in Figure 9, at each time step, the agent observes the current state S_t_, selects an action a_t_, and receives the next state S_t+1_ and reward R_t+1_ from the environment. This feedback indicates whether the previous action improved swimming performance. The robotic fish gradually updates its policy and learns optimal movement patterns.

The primary objectives of current RL applications in RLFRs include adaptive maneuvering, trajectory tracking, station keeping, and swimming efficiency optimization under varying hydrodynamic conditions. Existing studies have predominantly adopted Q-learning, Deep Q-Networks (DQN), and Deep Deterministic Policy Gradient (DDPG), largely due to their early availability, conceptual simplicity, and successful demonstration in low- to moderate-dimensional control spaces. These methods allow RLFRs to autonomously adapt swimming patterns through online or offline learning, exhibiting robustness to flow disturbances and parametric uncertainty.

The typical RL algorithms employed in RLFR research are summarized in Table 2. It is worth noting that the focus on Q-learning, DQN, and DDPG reflects the historical development of learning-based control in robotic fish, rather than the broader landscape of modern reinforcement learning. To further facilitate cross-cutting analysis, a comparative synthesis table of representative RL-enabled robotic fish studies (Table 3) is presented. It mainly focuses on systematic relationships among joint complexity, sensing strategies, experimental fidelity, and recurring methodological limitations. Details will be discussed in the following subsections.

### 4.1. Q-Learning Algorithms

Q-learning is a model-free reinforcement learning (RL) algorithm that is an off-policy temporal-difference method. This approach maintains and updates a state-action-value function, Q(s, a), that quantifies the expected cumulative reward for taking action a in state s, and subsequently follows an optimal policy. This process occurs through iterative trial-and-error without the need for an explicit mathematical model of the environment [65,71]. For applications such as robotic fish, Q-learning offers significant advantages, as the underlying physics of fluid–body interactions are highly complex and analytically challenging to model [87]. During robotic fish interaction with the surrounding fluid, the learning process is evaluated based on a reward r. This reward enables the agent to assess the effectiveness of the current action and updates the Q-values using the standard off-policy update rule. Q-learning has been successfully deployed for several complex tasks: coordination swarm control [65], avoiding obstacles and keeping a distance from the wall [74], adaptive thrust optimization [75], heading control and trajectory stabilization [76], and straightforward motion [77].

**Figure 10:**
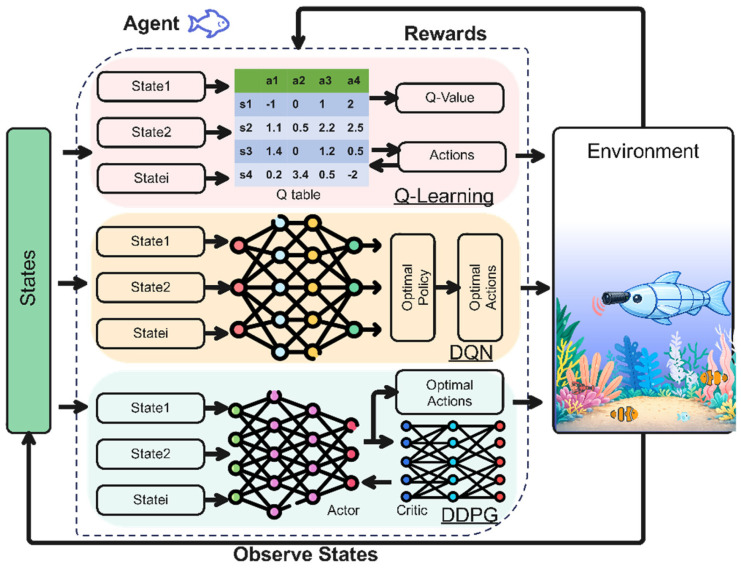
Comparison of Reinforcement Learning Architectures for RLFR Control.

In Q-learning, data refers to a multi-dimensional array of biological, sensory, and computational information used to design, control, and evaluate robotic fish using a Q-table, as shown in Figure 10 (top). RLFR data collection relies on multiple sensors that measure physical states and environmental parameters in real-time. Commonly used sensors include inertial measurement units (IMUs) for orientation, magnetometers for heading estimation, pressure sensors for depth, and flow or force sensors near the tail for hydrodynamic feedback.

Additionally, high-speed cameras and optical tracking systems are often used in laboratory setups to record body kinematics and aid in reward computation. In underwater environments, measurements are often corrupted by flow-induced noise, actuator backlash, and sensor latency, necessitating that RL states be low-dimensional, temporally averaged, or discretized to maintain learning stability. Consequently, many early RLFR studies favored simple sensing pipelines that strike a balance between observability and onboard computational limitations.

For instance, Liu et al. [74] implemented obstacle-avoidance and wall-following tasks using infrared (IR) sensors mounted around the robotic fish to detect nearby obstacles and tank walls by measuring reflected IR intensity. The behavior layer converted these measurements into fuzzy sensory states, which were then sent to a cognitive layer employing a Q-learning module for adaptive decision-making. Chen et al. [75] mounted an IMU at the robotic fish’s center of gravity to record the swimming heading angle, which acted as the real-time state input for the Q-learning algorithm. The average heading angle after each beat cycle was used to define the observation, which was discretized into state categories for table-based learning. This research highlighted a fundamental practical difficulty for underwater reinforcement learning. The delay of the Inertial Measurement Unit (IMU) and disruptions caused by water movement required using observations averaged over a cycle rather than instantaneous data. This approach prioritized learning robustness above immediate response.

Subsequently, Chen et al. [76] attached a six-axis load cell to the caudal fin to directly measure hydrodynamic thrust at a rate of 200 Hz. The average thrust over multiple flapping periods was discretized into 11 force-based states (0–0.48 N) and paired with five discrete amplitude-adjusting actions (−5°, −2°, 0°, +2°, +5°). The Q-table was updated in real time on a 72 MHz STM32 microcontroller, demonstrating on-hardware learning under strict memory, latency, and power constraints without reliance on simulation. While standard Q-learning typically requires a discrete state and action set, robotic fish often need to be operated in continuous underwater environments. Researchers [65,87] often employ fuzzy logic techniques to discretize variables such as position, orientation, and distance. By converting these continuous variables into discrete categories, the robot can store learned behaviors in a limited memory space. Despite this discretization, the system retains the ability to respond to the subtle variations inherent in continuous movement. This approach supports efficient memory use and facilitates effective learning in complex environments.

From the perspective of action-space design, Q-learning-based robotic fish reinforcement learners (RLFRs) typically utilize indirect control representations. In this framework, the actions selected by the agent correspond to high-level oscillation parameters, such as tail-beat amplitude, tail-beat frequency, or incremental parameter adjustments, rather than commanding direct joint torques or specifying continuous joint angles. This indirect approach to action selection helps to reduce the system’s sensitivity to sensor noise and actuation latency. Furthermore, it significantly lowers the dimensionality of the action space, which is essential for achieving stable convergence in tabular Q-learning methods.

The connection between mechanical design and RL complexity becomes evident when comparing single-joint and multi-joint RLFRs. Single-joint robots typically expose only one or two control variables (e.g., tail beat amplitude and frequency), allowing Q-learning to converge rapidly with simple reward functions. In contrast, multi-joint RLFRs introduce higher-dimensional action spaces, stronger inter-joint coupling, and increased sensitivity to timing and phase coordination, significantly expanding the search space and slowing convergence for tabular methods. As the number of joints and degrees of freedom increases, discretized Q-learning becomes impractical due to exponential state–action space growth, leading to continuous control and actor–critic methods. Effective reward design is crucial for stable robotic fish swimming; cycle-averaged metrics like mean thrust or energy use are preferred over noisy, delayed sensor data.

In summary, Q-learning provides a straightforward, model-free, and computationally efficient framework for controlling robotic fish in complex underwater environments, enabling the learning of adaptive and deliberative behavior without explicit hydrodynamic modeling. However, Q-learning-based RLFRs struggle to generalize beyond narrowly defined conditions. Training can be slow, performance is sensitive to the discretization of states and actions, and scalability rapidly degrades as robot morphology becomes more complex. These challenges motivate the shift toward function-approximation methods, sim-to-real learning pipelines, and energy-aware control strategies.

### 4.2. Deep Q-Network Algorithms

The Deep Q-Network (DQN) algorithm is an extension of classical Q-learning that uses a deep neural network to handle nonlinear and unstable flow environments, as illustrated in Figure 10 (Middle). The robotic fish functions as an intelligent agent capable of learning path-planning and obstacle-aware navigation [77], coverage path planning and motion optimization [77,78], environmental monitoring [91], gliding-motion efficiency optimization [79], formation control [80], and collective behavior or schooling modeling [81]. The objective is to approximate the optimal action-value function Q (s, a) that maximizes the expected discounted cumulative reward. In DQN, the tabular Q (s, a) is replaced by a deep neural network parameterized by weights and trained using stochastic gradient descent to minimize the temporal-difference (TD) error [77,78,79,80,81,82,83,84,91].

Tian et al. [91] proposed three mechanisms for improving DQN performance:

(1) A dynamic integrated reward that encourages efficient goal-directed movement while adapting to currents and avoiding collisions.
(2) A two-step action-selection strategy that starts with Boltzmann exploration and gradually transitions to ε-greedy action selection.
(3) A double-level dynamic learning rate that combines meta-gradient adjustment with Adam optimization for faster and more stable training.

Using this approach, robotic fish directly learns heading and velocity commands from sensory inputs.

Lin et al. [78] developed a DQN-based coverage path planning (CPP) algorithm for an RLFR. Onboard sensors, such as GPS and IMU, measured position and orientation, while pressure sensors estimated depth, and multi-parameter probes collected water-quality information. Joint angles and thrust were monitored using encoders and load cells, while obstacle and boundary awareness were provided by vision or sonar sensing. This sensor configuration reflects a common engineering trade-off in underwater RL systems: increasing state observability improves learning performance but also amplifies sensor noise, latency, and onboard computational load. For sim-to-real transfer, the DQN policy was first trained in a Python Gym-based grid environment and subsequently fine-tuned on real hardware using experimentally measured hydrodynamic response data, demonstrating the effectiveness of incremental realism and hardware-in-the-loop refinement for bridging the reality gap.

Similarly, Ma et al. [80] employed gyroscopes, accelerometers, and pressure sensors to measure heading, pitch, and depth, while three underwater cameras recorded swimming trajectories, and their controller was explicitly deployed and evaluated in real-world experiments. The reward function incorporated both energy consumption and trajectory deviation to enable continuous self-learning from sensor data. To improve robustness to waves, currents, and sensor disturbances, the DQN controller was pre-trained in simulation with domain-randomized noise, turbulence, and sensor perturbations before real-world deployment, demonstrating a domain-randomization-based sim-to-real strategy. In contrast, several other studies in this body of work report performance only in simulation environments, often relying on high-fidelity hydrodynamic models without physical validation. Across the literature, reported sim-to-real techniques can be broadly categorized into (i) domain randomization during simulation training, (ii) fine-tuning or continued learning on physical robots, and (iii) transfer learning using experimentally derived hydrodynamic parameters. Additionally, hydrodynamic modeling using CFD and IB–LBM techniques has been integrated to create realistic training environments, reduce trajectory error, and support sim-to-real transfer even in studies lacking full hardware validation [79,80,81,82,83,84].

Overall, DQN extends traditional Q-learning by replacing the discrete Q-table with a deep neural network that approximates the high-dimensional, continuous state-action space. The primary advantage of DQN is its model-free, data-driven learning, enabling the robotic fish to adapt without requiring an explicit mathematical hydrodynamic model. Continuous state observation, encompassing velocity, thrust, and orientation, enables the agent to optimize its actions through trial and error. DQN also provides scalability across different robotic morphologies and environmental conditions, making it suitable for multi-joint robotic fish. However, DQN requires substantial training data and computational resources, especially in high-fidelity simulations, and Sim-to-Real transfer remains challenging due to sensor noise, latency, and unpredictable hydrodynamic disturbances.

### 4.3. Deep Deterministic Policy Gradient Algorithms

As joint complexity rises, small control errors can propagate along the body. This leads to non-linear body–fluid interactions, which, in turn, increase exploration noise, slow convergence, and raise policy variance. Therefore, higher degrees of freedom practically necessitate actor–critic methods, such as DDPG, which are better suited to continuous, high-dimensional coordination across joints. Unlike discrete algorithms such as Q-learning or DQN, Deep Deterministic Policy Gradient (DDPG) algorithm handles continuous control of joint angles on RLFRs through parameters inherently continuous in aquatic locomotion. From an action-space design perspective, DDPG allows the controller to act directly on joint-level variables, such as joint angles, angular velocities, or torques, rather than adjusting high-level oscillation parameters as is common in tabular or value-based methods. This direct form of control becomes increasingly important as the number of joints increases, because precise coordination among multiple joints is difficult to achieve using only a small set of abstract oscillation parameters. The DDPG design framework generally consists of an actor-critic architecture, as illustrated in Figure 10 (Bottom). The actor–critic architecture in the DDPG framework, where the agent (fish robot) is composed of the Actor (Policy) and the Critic (Value Function). The Critic updates its estimation accuracy, while the Actor uses this feedback to refine its policy toward actions that maximize long-term performance [85,86,87,88,89,90]. Xu et al. [86] developed a DDPG controller integrated into a two-joint rigid-link bionic fish robot to perform an energy-efficient propulsion task. Joint encoders embedded within the servomotors provided continuous measurements of angular position, velocity, and acceleration for both joints, forming a compact yet expressive state vector. In parallel, current and voltage sensors recorded instantaneous electrical power consumption at each joint. These signals were filtered onboard and transmitted to a microcontroller, which computed propulsion efficiency as the ratio of useful hydrodynamic work to total mechanical energy input. The resulting state–action–reward tuples were stored in an experience replay buffer for off-policy learning. Importantly, the two-joint configuration provided sufficient actuation redundancy, allowing DDPG to fine-tune inter-joint coordination while keeping the action space low-dimensional enough to avoid unstable critic estimation. This resulted in smoother power output and a reported 9.77% increase in propulsion efficiency.

Vu et al. [89] created a robotic fish equipped with a camera stabilizer and a DDPG-based controller for underwater target tracking and, importantly, validated the learned policy on real robotic hardware rather than limiting evaluation to simulation. Two IMU sensors are mounted on the fish body and the camera to measure and compensate for yaw motion during swimming. Meanwhile, the camera’s sensor captures underwater images, which are then processed using the Kernelized Correlation Filter (KCF) algorithm to determine the target’s location within the frame. The DDPG was first trained in a simulation environment with a reward function. After approximately 20,000 training steps, the algorithm converged with a total reward approaching its optimum, and the simulated system achieved a rapid 5 s transient response without oscillation. To enable stable deployment on the physical robot, the authors explicitly addressed sim-to-real discrepancies by incorporating domain randomization and adaptive replay during training, mitigating hydrodynamic variability and sensor latency. As a result, the real-world experiments demonstrated stable tracking performance even under static bias (β_offset = 0.2) and time-delay disturbances, confirming successful sim-to-real transfer.

Yu et al. [90], by contrast, focused primarily on real-world data-driven learning rather than simulation-based validation, implementing a two-stage DDPG algorithm within a two-stage transfer learning framework to enable a four-joint robotic fish to learn swimming behaviors directly from biological fish motion data. In the first stage, biological motion data were collected from a snakehead fish using a custom motion-capture setup that included a glass tank and a GoPro 9 camera, which recorded at 1080p resolution and 100 Hz. A deep-learning-based skeleton extraction tool, DeepLabCut, was employed to identify six key points (head, joints 1–4, and tail) from the video frames using a trained ResNet50 network. This approach effectively bypassed conventional sim-to-real transfer by grounding the learning process in real biological motion, although the resulting controller was not explicitly validated across varying hydrodynamic conditions. Overall, the DDPG algorithm represents a significant advancement for RLFRs that require smooth and continuous control. However, the algorithm’s performance is strongly constrained by the complexity of fluid–body interactions, as transfer-based approaches often lack robustness to hydrodynamic variability and actuator–structure mismatches.

While DQN and Q learning often cause jerky or unstable tail-beat movements (limited to discrete actions) on RLFRs, the DDPG algorithm produces continuous action outputs and fine-tunes joint angles via the actor network, which directly maps sensory states to control signals. Additionally, DDPG learns policies directly via gradient-based optimization rather than searching discrete action spaces, as in DQN and Q-learning, leading to faster adaptation. Meanwhile, real-world underwater sensing, particularly from IMUs and pressure sensors, is subject to vibration, flow-induced noise, and latency, all of which can destabilize gradient updates if not properly filtered or regularized. These challenges motivate emerging extensions such as TD3, SAC, and hybrid model-based RL approaches. However, the fact is that the advanced algorithms, such as Proximal Policy Optimization (PPO), Soft Actor-Critic (SAC), and Twin Delayed DDPG (TD3), have demonstrated improved stability, enhanced exploration, and reduced sensitivity to hyperparameters. Their use in RLFR applications is still relatively limited. Similarly, transformer-based models have not yet been widely investigated for robotic fish control. They can be a potential direction for future research.

### 4.4. Reward Configuration Strategies and Design Challenges

In RL for robotic fish, the reward is typically a scalar feedback signal that evaluates the controller’s actions and guides the learning process toward an optimal policy [71]. To accomplish their targeted tasks, robotic fish often need to balance forward motion, stability, and energy efficiency. As a result, reward configuration strategies often combine multiple objectives to promote desired physical behavior while penalizing unfavorable ones.

Based on the existing literature, reward configuration strategies for robotic fish control can be broadly categorized into four types. The first focuses on velocity and attitude tracking, aiming to maintain a target speed and minimize heading or orientation errors, a formulation closely related to the so-called flapping paradox [71]. The second emphasizes thrust-generation efficiency, particularly in policy search algorithms applied to caudal-fin actuation to maximize propulsion efficiency [92]. The third category addresses swarm and formation control, where rewards are designed to coordinate collective behavior in multi-agent scenarios [25,26]. The fourth involves event-driven rewards in game-based or competitive tasks, where discrete success or failure events define the reward structure [65].

Early Q-learning and DQN-based approaches typically rely on velocity-tracking or efficiency-oriented rewards combined with simplified control parameters, which helps mitigate the impact of sensor noise and communication delays. However, because these methods operate with discrete action spaces, they often produce jerky tail motions, unstable oscillations, or energetically inefficient swimming patterns. As the number of joints increases, reward functions that overly prioritize speed can further exacerbate poor inter-joint coordination and excessive energy consumption. Continuous-control methods such as DDPG enable smoother, more biologically realistic swimming motions. However, their performance remains highly sensitive to rewarding scaling and to the careful balancing of multiple competing objectives in the presence of unobserved flow and vortex dynamics. The systems can appear non-Markovian to the agent/robotic fish, making the reward signal noisy and inconsistent, which destabilizes learning. To address this challenge, transformer-based models are increasingly being explored as potential solutions for mitigating apparent non-Markovian effects by exploiting long-range temporal dependencies to capture latent hydrodynamic dynamics.

## 5. Discussion

This section discusses key challenges and future trends in two parts, with the goal of providing practical insights and potential directions for advancing the future development of RLFRs in mechanical design and RL control.

### 5.1. Challenges

#### 5.1.1. Challenges in Design

The RLFRs have demonstrated the development from the early single-joint prototypes to later multiple-joint designs. However, some limitations remain in achieving a fully biomimetic and efficient underwater locomotion system. The first main challenge is waterproofing. RLFRs generate thrust via the movement of active joints, requiring complex sealing with O-rings, silicone gaskets, or epoxy encapsulation. But these solutions often increase bulk, restrict flexibility, and cause mechanical wear during long operations. Integrating more sensors without trading the integrity of the seal remains an unresolved issue. The ability to transmit torque and achieve synchronization efficiency is another limitation. Multi-joint RLFRs typically use servo motors at each joint, so mechanical backlash or time delays between joints can distort traveling-wave propagation, leading to inefficient thrust generation and irregular tail amplitudes. Moreover, a decrease in amplitude at higher frequencies due to hydrodynamic loading along the body reduces the achievable speed-to-power ratio. These impacts are clearly observed when the fish robot works in unsteady flow, making precise phase coordination difficult to maintain. Another challenging factor in Biomimetic underwater vehicles (BUVs), including RLFRs, is energy management. Most RLFRs operate at limited capacity, resulting in short operating times (typically below 1 h). The propulsion of RLFRs relies on multiple servo motors, which increase power consumption and limit long-term missions, such as monitoring or exploration, in the marine environment. Additionally, material and structural trade-offs directly impact hydrodynamic performance. For example, rigid frames made from aluminum or carbon fiber provide strong actuation transmission but lack compliance, while flexible segments made of silicone or polymers increase flexibility but have dimensional instability in water. Lastly, reinforcement learning integrated into the controller of RLFRs requires many sensors. This directly increases weight and shifts the center of mass, leading to structural instability.

#### 5.1.2. Challenges of Reinforcement Learning

An RL has been widely integrated into controllers of RLFRs. Yet, it still faces several critical challenges that affect stability, adaptability, and efficiency. First, the difficulty of transferring RL policies trained in simulation to real aquatic conditions. Most training environments use simplified hydrodynamic models that cannot accurately capture the nonlinear effects in real swimming, including viscous drag, vortex shedding, and unsteady wake interactions. These unmodeled dynamics lead to the differences between simulated states and sensor feedback, resulting in reduced control accuracy and unstable motion. Moreover, physical disturbances in the marine environment, such as small-scale turbulence or flow asymmetry, are impossible to replicate in simulation.

Second, the RL algorithms summarized previously require millions of interactions between the agent and the environment to converge on optimal behavior. Therefore, experiments are time-consuming, energy-intensive, and costly to operate. Additionally, the transmission of sensor data wirelessly through water to the controller is often slow, which can prolong experiments. As a result, most studies rely on simulation-only training, which reduces data realism and limits robustness.

Third, the above RL algorithms have high computational throughput and large memory requirements, which exceed the capabilities of the small microcontrollers typically used in RLFRs. Therefore, the processing delays have often occurred, destabilizing control and slowing the learning process. Additionally, external processors designed to enhance the microcontroller’s computational capabilities often experience communication failures in water.

Fourth, underwater environments are inherently uncertain due to constantly changing flows, unpredictable turbulence, fluctuating pressures, and sensor noise, which can distort and often mislead the RL agent during policy updates. For instance, a sudden vortex or sensor drift may be misinterpreted as a failed action, causing the network to update incorrectly. Furthermore, water opacity and refraction degrade visual sensing, reducing the reliability of image-based reinforcement learning approaches.

Fifth, the continuous learning process requires sustained computation, which rapidly drains battery power and causes internal heating. In compact systems such as the fish rigid head, heat dissipation is limited, leading to thermal stress on processors and actuators.

### 5.2. Future Directions

#### 5.2.1. Future Directions for Physical Platforms and Designs

Future directions for the physical platforms and designs of robotic fish are centered on high-dimensional, modular, and reconfigurable systems, hybrid rigid-flexible structures, and sustainable, silent, and energy-autonomous designs.

One of the major future directions is to prioritize high-dimensional, modular, and reconfigurable systems specifically engineered to facilitate rapid prototyping and reconfiguration. As discussed, multi-joint systems fundamentally improve controllability and maneuverability, but they are more vulnerable to joint backlash, phase mismatch, and hydrodynamic uncertainty. To address those issues, future physical/mechanical platforms and designs that prioritize high-dimensional, modular, and reconfigurable designs are necessary, including precise joint sensing, reduced transmission compliance, simplified drive mechanisms, and modular waterproof structures that facilitate scalable experimentation, exploration, surveillance, and research on aquatic ecosystems.

Another promising future direction is to enable active variable stiffness and hybrid rigid–flexible designs to reconcile actuation authority with passive dynamics in RL-enabled robotic fish. Such designs and structures can decrease the control effort necessary for propulsion and maneuvering by enabling certain body segments to store and release energy through elastic deformation. When integrated with machine learning and deep learning, compliant elements can transform from modeling uncertainties to exploitable degrees of freedom that adaptive learning policies might utilize under varying flow conditions.

Driven by the need for “Net Zero” oceanographic capability, a complementary future direction is the development of sustainable, silent, and energy-autonomous designs. Silent propulsion is essential for sustained oceanographic monitoring, prompting the development of vibration-free actuation technologies like dielectric elastomers. Concurrently, renewable energy sources, such as solar power, microbial fuel cells, and sophisticated seawater batteries or supercapacitors, are being investigated to facilitate continuous operation. The emphasis on using eco-friendly, biodegradable materials is growing to reduce environmental impact during extended deployments in delicate maritime ecosystems. Future iterations will focus on improving the life cycle and mobility of robotic fish.

#### 5.2.2. Future Directions for Control Strategies

Although modern continuous-control reinforcement learning algorithms such as Proximal Policy Optimization (PPO), Soft Actor-Critic (SAC), and Twin Delayed Deep Deterministic Policy Gradient (TD3) have shown strong performance in many robotic locomotion problems, their use in rigid-link robotic fish remains relatively rare. In principle, these methods are well-suited to robotic fish control. They can generate smooth, continuous joint motions, maintain stable learning behavior, and better handle uncertainty, all of which are important for underwater swimming, where motion is oscillatory, nonlinear, and affected by noise and disturbances. In practice, however, several factors limit their adoption. First, algorithms such as PPO and SAC typically require many training interactions to converge. Conducting these interactions underwater is expensive, slow, and energy-intensive, making extensive real-world training impractical. Second, these algorithms are computationally demanding. For example, SAC relies on multiple neural networks and entropy tuning, which can exceed the processing and memory capabilities of the embedded controllers commonly used in robotic fish. Third, continuous-control policies are highly sensitive to differences between simulation and real environments. Small mismatches in hydrodynamic forces, actuator behavior, or sensor delays can significantly degrade performance when transferring learned policies to physical robots.

As a result, many existing studies have preferred simpler methods such as DDPG, which offer a practical compromise between control flexibility and implementation complexity. Future research could overcome these limitations by combining reduced-order control representations, physics-informed learning, and offline or transfer learning approaches to make advanced algorithms more feasible for rigid-link robotic fish.

To reduce the gap between simulation environments and real-world hydrodynamics, physics-informed reinforcement learning (PIRL) should be employed. PIRL embeds analytical fluid-dynamics equations or drag-force models directly into the neural network’s training objective, enabling agents to learn more efficiently while respecting physically consistent constraints. At the same time, the RL agent can be trained to generalize across uncertain real-world conditions through domain randomization, which stochastically varies fluid density, viscosity, and turbulence intensity during simulation. In addition, future studies may incorporate internal predictive models to estimate future states, thereby reducing the number of physical experiments required. Another viable strategy for RLFRs is transfer learning, in which policies trained on one robot morphology or environment are adapted to another via weight initialization and fine-tuning, thereby avoiding redundant training cycles. Furthermore, RL algorithms may adopt network architectures with fewer hidden layers or utilize spiking neurons to lower computational demands while preserving adaptive behavior. To improve robustness under uncertain underwater conditions, sensor fusion and uncertainty-aware learning frameworks should be integrated into the next generation of RLFRs. In this way, the agent can achieve more stable state estimation by combining vision, flow, pressure, and IMU data using Kalman or particle filtering.

Through these coordinated developments, future research should prioritize training RLFRs to accomplish high-level autonomous tasks, including dynamic path planning and obstacle avoidance in cluttered coral reef environments, cooperative schooling or formation control for distributed ocean monitoring, and adaptive sampling missions in which multiple robots collaboratively map temperature, salinity, or pollutant gradients.

Moreover, from a learning standpoint, soft robotic fish and RLFRs constitute complementary paradigms, emphasizing embodied compliance and joint-level control, respectively. As part of future work, soft robotic fish may be further investigated through hybrid rigid–soft designs, where reinforcement learning coordinates joint-level control with compliant, energy-efficient body deformation.

## 6. Conclusions

This review has provided a comprehensive and systematic synthesis of rigid-link fish robots (RLFRs) with a particular focus on the interplay between mechanical design, sensing, and reinforcement learning-based control. By examining more than two decades of literature under the PRISMA framework, we highlighted how increasing articulation—from single-joint to multi-joint architectures—fundamentally reshapes controllability, learning complexity, and achievable swimming performance in biomimetic robotic fish.

From a mechanical standpoint, rigid-link architecture offers a compelling balance between structural simplicity and expressive locomotion. Single- and two-joint RLFRs serve as effective platforms for validating hydrodynamic principles and early reinforcement learning strategies due to their low-dimensional and interpretable control spaces. As joint count increases, three-joint and multi-joint designs enable smoother body-wave propagation and more biologically realistic carangiform swimming, but at the cost of increased coordination complexity, higher energy demand, and more challenging waterproofing and actuation requirements. Compared with soft robotic fish, RLFRs provide deterministic kinematics, clearer state observability, and improved repeatability, making them particularly well suited for data-driven learning and sim-to-real transfer.

From a control perspective, reinforcement learning has demonstrated clear advantages over classical model-based and CPG-based approaches by enabling adaptive, model-free control in the presence of complex, uncertain fluid–structure interactions. Q-learning, DQN, and DDPG remain the most widely adopted algorithms in RLFR research, primarily due to their conceptual simplicity and early success in low- to moderate-dimensional control problems. However, our review reveals that these methods are often applied as parameter-tuning tools rather than as enablers of fundamentally new behaviors. Common limitations—including low sample efficiency, sensitivity to reward design, instability during training, and poor generalization across platforms—continue to constrain their broader adoption in real-world underwater environments.

A key insight emerging from this review is that reinforcement learning performance in robotic fish cannot be decoupled from mechanical design and sensing strategies. Joint architecture directly influences the action space structure, state observability, and reward formulation, thereby shaping the feasibility and scalability of learning-based control. As articulation complexity increases, the benefits of reinforcement learning become more pronounced, but only when supported by appropriate sensing, a wide actuation bandwidth, and an energy-aware design. This highlights the need for system-level co-design approaches that jointly consider morphology, control, and learning, rather than treating reinforcement learning as an isolated algorithmic component.

Several research directions are critical for advancing RL-enabled RLFRs, including improving sim-to-real transfer through domain randomization and physics-informed learning, integrating hybrid active–passive joint designs to reduce control burden, adopting more modern and stable RL algorithms, and developing morphology-aware learning frameworks that generalize across different robot designs. Furthermore, multi-objective learning that explicitly balances speed, efficiency, robustness, and task performance, as well as cooperative learning among multiple robotic fish, represents a promising avenue for future exploration.

## Figures and Tables

**Table 1:** Summary of biomimetic robotic fish configurations and performance.

Type	Length/Speed	Material	Joint Position/Actuation	Energy Efficiency (with Duration)	Reliability	Cost (Approx., Representative Systems)	Key Limitations
**Single-joint** [2,13,25,26,27,28,29,30,31,32,33,34]	5–50 cm; typically ≤1.5 BL/s	Polymer body (ABS/PLA); simple plastic or Mylar caudal fin	Single actuated joint at caudal peduncle; small servo or DC motor	Low (~20–90 W); tail-only oscillation with high slip losses; >24 h continuous operation	High; minimal mechanical parts; simple sealing; robust for long-term operation	$200–$600 (educational/interactive robotic fish; embedded-vision guided single-tail systems)	Overly simplified hydrodynamics; inefficient steady swimming; weak maneuverability; unsuitable for studying body–wave dynamics
**Two-joint** [3,34,35,36,37,38,39,40,41,42,43,44,45]	30–50 cm; ~0.8–2.5 BL/s	3D printed polymer body; aluminum or polymer links; silicone tail	Two serial joints at mid-body and tail base; geared servos	Medium (up to 100 W); partial body deformation improves thrust; ~1–3 h operation	Medium–High; moderate mechanical and sealing complexity; stable in lab tests	$800–$1800 (two-joint laboratory platforms for CPG, antiphase, and formation studies)	Body wave remains discontinuous; limited transport efficiency; scaling to complex swimming patterns constrained
**Three-joint** [4,46,47,48,49,50,51,52,53,54,55,56,57,58,59]	40–70 cm; ~0.9–3.0 BL/s	Waterproof polymer shell; reinforced metal or composite joints; elastomeric tail	Three serial joints forming posterior body wave; servo or electromagnetic actuators	Medium–High (~100 W); closer to carangiform BCF motion; ~1–2 h operation	Medium; sensitive to phase mismatch, backlash, and joint sealing degradation	$2000–$4500 (ICHTUS 3-DOF fish; Fibo-series prototypes; environmental monitoring fish)	Control tuning becomes critical; performance strongly phase-dependent; learning-based control stability decreases
**Multi-joint** [6,10,60,61,62,63,64,65,66,67,68,69,70]	50–125 cm; ~1.0–4.0 BL/s	Modular sealed body segments; aluminum or carbon-fiber frame; flexible caudal fin	≥4 distributed hinge joints along flexible tail; one actuator per joint	High (>150 W); near-continuous traveling body wave yields best transport efficiency; ~0.5–2 h operation	Medium–Low; increased actuator count, sealing points, and wiring raise failure probability	$5000–$12,000+ (four-joint carangiform robotic fish; electromagnetic multi-joint fish; leader–follower platforms)	High-dimensional control space; strong hydrodynamic coupling; high computation and calibration cost; sensitive to noise and sim-to-real gap

Note: The reported operation durations correspond to typical continuous swimming or task execution times observed in experimental studies, rather than maximum battery endurance.

**Table 2:** Summary of reinforcement learning algorithms applied to the RLFRs.

Algorithm	Policy/Model	Learning Task	Sensors Used	Performance Tasks
**Q-Learning** [65,74,75,76,77,78]	Off-policy & model-free	Optimal value function	IMU; IR proximity sensor; pressure sensor; visual tracking camera	Heading stabilization; thrust optimization; cooperative swimming; obstacle avoidance
**DQN** [79,80,81,82,83,84]	Off-policy & model-free	Optimal value function	IMU; pressure sensor; water-current sensor; visual tracking camera	Path planning; formation control; energy-efficient navigation; trajectory tracking
**DDPG** [85,86,87,88,89,90]	Off-policy & model-free	Continuous control via actor–critic policy learning	IMU; pressure sensor; hydrodynamic force sensor; visual tracking camera	Propulsion-efficiency optimization; adaptive stiffness control; trajectory tracking; energy-efficient navigation; target tracking

**Table 3:** Comparative synthesis of RL-enabled robotic fish studies.

	Reference	Joint Architecture	Target Task	Sensors	Continuous Control Variables	Evaluation Metrics	Experimental Conditions	Limitations
**Q-learning**	Yu et al. [65]	Multi-joint robotic fish (3 joints)	Cooperative behavior learning	Vision-based global tracking; posture estimation	Linear speed ω; angular speed ω (via fuzzy inference)	Cumulative reward; time steps to win; task success rate	Real-world pool experiments (2 vs. 2 robotic fish competition)	Discrete state/action spaces; learning relies on vision; centralized control
	Linet et al. [77,78]	Single-body rigid bionic robot (no joints)	Heading control	Virtual (simulation-based)	Fin waveform parameters (amplitude, frequency)	Tracking stability; learning efficiency; motion accuracy	Numerical simulation	Early feasibility study; no physical sensing reported
	Chen et al. [75,76]	1-DOF robotic fish	Motion control	No sensors	CPG neutral position; flapping parameters	Heading accuracy; stability; robustness	Simulation + prototype	Used mainly for feasibility validation; no sensing-based learning
**DQN**	Chen et al. [81]	Abstract fish-schooling agents	Collective behavior	Relative-position sensing	Discrete heading-angle change mapped to continuous velocity update	Inter-agent distance error; polarization order; collision avoidance rate	Multi-agent numerical simulation	Agents rely on relative distance/angle; states derived in simulation
	Sun et al. [84]	Tail-driven robotic fish (implicit single-joint)	Formation control	Virtual position and velocity sensing	Discrete heading and speed commands mapped to tail-beat motion	Formation error; inter-agent distance deviation; convergence time	CFD-based simulation with imitation learning	State variables derived from CFD flow field and relative positions
	Tian et al. [91]	Low-DOF bionic robotic fish	Path planning	Virtual flow-field and position sensing	Heading and speed under ocean currents	Path length; travel time; path smoothness	Simulation with ocean current disturbances	Ocean-current effects are explicitly modeled; sensing is environment-defined
**DDPG**	Ma et al. [80]	Hybrid-driven robotic fish (~2–3 DOF)	Path tracking	IMU (orientation); velocity estimation (simulation)	Heading angle; pitch angle; continuous DDPG outputs	Absolute tracking error (XYZ); MSE; MAPE; path-following accuracy	Dynamic simulation	Continuous states include pose and velocity; no physical sensors used
	Duraisamy et al. [85]	Multi-joint robotic fish	Speed tracking	IMU; joint encoders; motor sensors	Tail oscillation amplitude & frequency	ISE; IAE; ITAE; speed tracking error	Data-assisted model; real experiments	Experimental platform reports onboard sensing
	Xu et al. [86]	4-joint robotic fish	Propulsion control	Virtual hydrodynamic force sensing	Joint stiffness (continuous modulation)	Propulsion efficiency; power consumption	High-fidelity CFD simulation	Physics-based sensing variables extracted from CFD solver
	Cui et al. [73]	Two-joint bionic robotic fish	Propulsion efficiency optimization	IMU; joint angle sensors; thrust estimation	Joint angle increments; body lateral displacement	Thrust coefficient; propulsion efficiency; power consumption; reward	Simulation + real-world experiments	Sensors used for closed-loop stiff-ness optimization experimentally
**Enhanced DDPG**	Vu et al. [89]	Elongated undulating fin robot	Swimming motion optimization	Virtual fin-ray state sensing; force estimation	Oscillatory amplitude of H-CPG for each fin ray	Thrust; propulsive efficiency; accumulated reward	Multi-agent CFD simulation + experiments	Each fin ray treated as an agent with local state feedback
	Yu et al. [90]	Four-joint bionic robotic fish	Sim-to-real control transfer	IMU; joint encoders; motion capture (offline)	Joint oscillation amplitudes & swing speed	Acceleration capability; swimming efficiency; maneuverability	Two-stage sim-to-real learning	Offline sensing data used to bridge sim–real gap

## Data Availability

Not applicable.
